# Funding innovation for treatment for rare diseases: adopting a cost-based yardstick approach

**DOI:** 10.1186/1750-1172-8-180

**Published:** 2013-11-16

**Authors:** Garret Kent Fellows, Aidan Hollis

**Affiliations:** 1 Department of Economics, The University of Calgary, 2500 University Dr. N.W, Calgary, AB T2N 1 N4, Canada

**Keywords:** Price regulation, Orphan drugs, Rate of return, Yardsticking

## Abstract

**Background:**

Manufacturers justify the high prices for orphan drugs on the basis that the associated R&D costs must be spread over few patients. The proliferation of these drugs in the last three decades, combined with high prices commonly in excess of $100,000 per patient per year are placing a substantial strain on the budgets of drug plans in many countries. Do insurers spend a growing portion of their budgets on small patient populations, or leave vulnerable patients without coverage for valuable treatments? We suggest that a third option is present in the form of a cost-based regulatory mechanism.

**Methods:**

This article explores the use of a cost-based price control mechanism for orphan drugs, adapted from the standard models applied in utilities regulation.

**Results and conclusions:**

A rate-of-return style model, employing yardsticked cost allocations and a modified two-stage rate of return calculation could be effective in setting a new standard for orphan drugs pricing. This type of cost-based pricing would limit the costs faced by insurers while continuing to provide an efficient incentive for new drug development.

## Background

Orphan drugs – those that treat small populations – are placing a substantial strain on the budgets of drug plans in many countries [1-4]. Prices commonly exceed $100,000 per patient per year and are increasing [5]. While these drugs offer important therapeutic benefits, prices are at a level that private and public insurers are struggling to manage. In many cases, insurers are faced with a deep quandary: how much are they willing to pay to save or extend a life by a few months or years? The reality of budget constraints for public insurance plans limits the amount that can be spent on drugs, orphan or not. The problem is likely to become more acute. Research is increasingly leading to more refined disease classifications so that potential and existing treatments face shrinking patient numbers [6,7].

The high cost of drug development, relative to total market size, is often cited by pharmaceutical firms to justify the high prices of orphan drugs. Firms argue that drug development costs are similar no matter how rare the disease or condition, but the number of patients for orphan drugs is much smaller, necessitating a high price for the firm to cover its costs. Current literature indicates that production cost and molecular complexity do not play a significant role in orphan drugs pricing; pricing is more directly correlated to rarity (drugs with a smaller patient group have higher costs) and “what the market will bear” [8].

Despite high prices, insurers continue to display some willingness to cover orphan drugs [1,9]. Given this preference and the fact that orphan drugs are associated with high average costs, the question arises: *is there an effective policy to limit the prices faced by drug plans while still providing sufficient incentive to pharmaceutical companies to develop and market orphan drugs?*

Without an effective standard on which to evaluate orphan drug prices, current policies are likely contributing to the problem of rising healthcare costs [7]. Other changes to the standard used to evaluate orphan drug pricing have been proposed. Some are modifications of the existing value-based or cost-effectiveness criteria [10-12]. An important analysis by Hughes-Wilson et. al. proposes an evaluation system that would depend on multiple parameters, including rarity, the extent of research undertaken, manufacturing complexity, and disease severity [13]. Our analysis could be seen as an input into a system such as the one they propose, or as offering an alternative.

Cost-effectiveness measures relate the price of a drug to a standardized measure of value, typically a QALY (quality-adjusted life year). Insurers and regulatory authorities commonly employ the “incremental cost-effectiveness ratio” to evaluate the price/coverage decision of new drugs [14]. For a given budget, drugs are ranked according to their cost effectiveness. Drugs with a cost-effectiveness above a specific threshold are then insured. The threshold is set to exhaust the given budget. Facing this system, firms tend to price to the expected threshold, and earn profits based on the differential between the threshold price and their costs of providing the product. Firms with the lowest costs of producing health generally earn higher profits.

Cost-effectiveness measures favor “common” drugs over orphan drugs due to the lower average cost of the former [2]. Realizing this, health and policy officials often make exceptions for orphan drugs; they may add a rarity premium to the cost/benefit measure, or use some other differential standard to justify the higher price of an orphan drug. The current application of these somewhat *ad hoc* policies are problematic as they do not replace the standard cost-effectiveness measure with any rule that helps to determine how much the insurer should be willing to pay for a given drug [1].

Given the high prices being paid, orphan drug development is becoming more profitable than development of drugs for common diseases [3]. The orphan drug market is currently growing faster than the market for traditional pharmaceuticals: 25.8% vs. 20.1% for the period 2001 to 2010 in the United States [15].

This suggests that the appropriate context in which to discuss orphan drug prices is to focus on a balance between sufficient compensation to firms that develop these drugs and control of the overall insurer budget [2]. Cote and Keating [16] note that many “orphan” drugs are financial blockbusters, and that the high prices justified on the basis of low prevalence become a path to excessive profits. Kanavos and Nicod [17] have proposed that it would be important to have a “benchmark” to determine whether the profitability of some drugs has been “excessive.” Our paper makes a start on establishing a basis for creating a benchmark.

**Figure 1:**
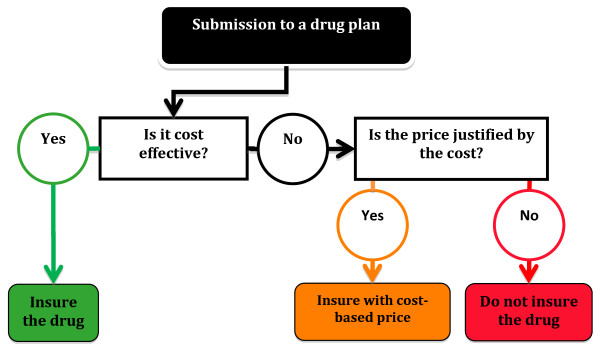
Proposed drug plan decision tree.

Since firms cite higher average costs in justification of higher orphan drugs prices, it seems natural that we consider a cost-based pricing approach. Cost-based pricing has not been seriously investigated as a pharmaceutical pricing solution, yet there is a substantial volume of literature on cost-based pricing in utilities where the model is widely employed [18]. The contribution of this paper is to exploit the utilities regulation literature to provide insight into the possibilities for cost-based regulation of orphan drugs prices. Based on comparisons to the regulatory mechanisms used in utilities sectors, we explore the use of a regulatory cost-of-service model modified for use by a government drug insurance plan. This could be implemented as the formal adoption of a cost-based approach in the price negotiation phase of approval. Should an orphan drug fail the cost effectiveness test (which is likely) we propose a cost-based mechanism to determine a “just and reasonable price” for the drug [19-21]. The prescribed approach is summarized by the flowchart below (Figure 1).

The remainder of this paper is laid out as follows: Section utilities as a model for orphan drugs: comparing cost structures examines similarities in cost structure between utilities and pharmaceutical firms as evidence that utilities regulation can inform orphan drugs pricing decisions. Section economic regulation: a primer with utilities case studies gives an overview of the rationale for economic regulation and describes two relevant models applied in utilities jurisdictions. Section drug price regulation details relevant existing economic interventions in pharmaceutical markets. Section rate of return model for orphan drug price regulation exposits our prescription for a cost-based pricing standard. Section conclusion, concludes.

### Utilities as a model for orphan drugs: comparing cost structures

Most utilities face a substantial “sunk” investment in the form of facilities construction (for example an electrical generation or pipeline facility). These costs are described by economists as “sunk” since, once constructed, these facilities have no alternative use and their value cannot be easily recovered through resale of purchased assets [22]. Most medical therapies share this characteristic of a substantial sunk cost in the form of research and development costs. Once R&D has occurred, the associated costs are not recoverable. Drug development costs have been estimated at between $207 million and $1.8 billion [23]. By comparison, the Canadian National Energy Board currently regulates pipelines with a range of established capitalized costs between $394 million and $6.17 billion.

In addition to the high sunk costs, the marginal cost of production (i.e. the cost to produce an incremental unit) tends to be low in both industries. In utilities industries, once a facility like a power-plant or pipeline has been constructed, small increases in the quantity of a good or service provided are associated with very small (or no) increases in cost. For a pipeline, as long as it is operating below its maximum capacity, an additional cubic foot or barrel of throughput does not have a substantive effect on the total cost of providing the service. The same is true in the production of drugs for rare diseases. Once a drug has been developed and approved, the cost of producing an additional unit is typically very small.

We can represent the average cost of production for a unit of output in either the orphan drug or utilities industries by equation (1):

(1) $$\mathrm{Average}\;\mathrm{Cost}=\frac{\mathrm{Fixed}\;\mathrm{Cost}}{\mathrm{Total}\;\mathrm{Quantity}}+\mathrm{Marginal}\;\mathrm{Cost}$$

Given the high fixed cost estimates cited above and the essentially negligible marginal cost for both drugs and utilities, the cost functions of both industries exhibit classic economies of scale. In both cases the average cost of production is falling as the total quantity increases. The addition of a second firm into such a market and the associated doubling of fixed costs will increase the average cost by splitting the same total quantity across twice the fixed costs. This fits a basic definition of a natural monopoly: an industry in which a single firm can serve the entire market at a lower cost than multiple firms [24].

In the pharmaceutical market it may be possible for a second firm to enter at a lower average cost by free riding on the incumbent’s research and development expenditure [25]. While the possibility of free-riding entrants may imply that pharmaceutical firms are not necessarily 'natural’ monopolies, patent protection (or other protections of exclusivity, including data protection) essentially give firms a government-granted monopoly [26]. The patent system has been set up with the express purpose of creating incentives for innovation, and the existence of patent laws creates an economic environment similar to a natural monopoly industry. Even when multiple drugs are approved for the same indication, the patent system is still present to restrict free entry. In this limited competition “oligopoly” type market, prices will continue to remain high absent external regulations.

An important difference between utilities and pharmaceutical companies costs concerns the timing of sunk expenditure. Historically, utilities incurred relatively little capital cost prior to regulatory approval of a new facility. By comparison, pharmaceutical firms must incur almost all of their sunk costs prior to applying for regulatory approval. This difference in the timing has implications for risk. In utilities jurisdictions, the productivity of fixed/sunk capital inputs (and associated expenditures) is relatively deterministic. That is, a firm or regulator can have a reasonable (and fairly accurate) expectation of how productive a capital input will be. In the pharmaceutical industry, fixed/sunk costs are generally associated with R&D expenditures, which by their very nature have an uncertain effect on revenue.

### Economic regulation: a primer with utilities case studies

In competitive markets (where there are several firms producing similar products), competition acts as a constraint on the price charged by any single firm. If one firm increases its price, consumers can switch to a lower priced substitute provided by a competitor. However, as discussed in the previous section most utilities and orphan drugs manufacturers are monopoly (or oligopoly) industries, in which a single firm (or a very small number of firms) can supply the entire market at a lower total cost than multiple (or a higher number of) firms. The introduction of an additional competing firm in a natural monopoly industry needlessly duplicates costs, potentially causing an increase in the market price and certainly reducing economic efficiency as measured by the difference between the total value of a product to consumers and its cost of production [24].

The unfortunate consequence of this is that standard competitive forces will not be present to limit the prices charged by firms in natural monopoly industries. This is why economic regulation is warranted and desirable. The goals of a desirable price control mechanism are twofold: i) to protect consumers from the market power of firms arising from an absence of competition and ii) to provide those firms with revenues sufficient to ensure fair compensation.

We present here an overview of two common models of economic regulation: yardstick and rate of return. These mechanisms form the basis for a substantial majority of existing economic regulation in the utilities and pharmaceutical sectors.

### Yardstick regulation

Yardstick regulation is a comparative pricing mechanism. In the traditional definition a regulator using this mechanism sets a regulated price for a firm’s product based on the costs incurred by similar firms producing the same good [27]. In conventional application regulators may use either the costs of similar firms, or observed prices in other jurisdictions or comparable markets in order to determine the 'fair’ price for a regulated good.

If there is a competitive industry on which to benchmark prices, then yard-sticking is a proxy for direct competition. Yardstick applications can also occur between two regulated markets wherein a regulator, observing a regulated price in another jurisdiction, will set the same price. This has the potential for circularity in regulatory mechanisms if two prices are benchmarked against each other, rendering the policy ineffective as a price control mechanism.

A key theoretical feature of yardstick regulation is the preservation of incentives for firms to reduce costs. Since the regulated price is not based on the firm’s own costs, the firm can increase profits by reducing costs. In pharmaceuticals, the yardstick approach is very widely used, but faces a significant challenge because the reference prices are often not the real prices paid, owing to the common practice of confidential rebates [4].

Yardsticking is also present in utilities jurisdictions like the electricity distribution sector. The Netherlands and Sweden as well as Chile regulate electricity distribution using a formal yardstick approach [28,29]. Italian water utilities are also regulated using a strict application of the original formulation [27] of the yardstick approach.

Conventional yardstick regulation is most common where there are several firms either operating in the same market, or operating in several similar markets where each firm has a regional monopoly. If there are very few firms in the market, or if firms are sufficiently dissimilar, direct application of the yardstick model becomes difficult or impossible as a realistic yardstick for a given firm will be difficult to identify [28].

### Rate of return regulation

Under rate of return (ROR) regulation the price is set such that it produces total revenues for the firm that are equal to a fixed and predetermined amount referred to as the “revenue requirement”. Application of ROR follows a multi-stage process. First, regulators aggregate the value of a firm’s assets into an account called the 'rate base.’ The regulator then estimates a 'fair’ rate of return based on the firm’s cost of capital. Often this is done using a formula in which some risk adjustment is added to a standard measure of borrowing costs (such as the long-term rate on government bonds) [30]. Elements of yardsticking can be incorporated into the determination of the regulated rate of return; regulated firms, their consumers and the regulator itself commonly reference (or yardstick) rates of return in similar unregulated industries.

The next step in ROR regulation is to add the product of the rate base and the determined 'fair’ rate of return to a measure of the firm’s variable costs (consumable materials like office supplies, advertising expenses or other intermediate input goods). A depreciation allowance is also added to the total, producing a “revenue requirement” adequate to compensate the firm for its costs and allow it to earn a fair return on its investments. Price is then set to achieve this revenue requirement.

Since the regulator is unlikely to have direct knowledge of the firm’s costs, there is often disagreement as to the costs included in the rate base. Determination of the firm’s cost of capital is also contentious due to the abstract nature of this value. The cost of capital can be described as the minimum return that investors require for providing capital; this is a value unobservable to anyone except the investors themselves.

Regulated prices are typically set for a fixed period. At the end of each period the regulator institutes a hearing to review cost and price elements and makes adjustments accordingly. This process usually follows a convention wherein the regulated firm and its consumers and interested parties are given the opportunity to present evidence on cost elements and the calculated rate of return.

Conventional application of the rate of return mechanism in a utilities setting includes a “used and useful” criteria on the allowable costs. Coupled with periodic regulatory hearings in which the regulated firm must defend its input decisions, the idea is to ensure that only relevant and necessary costs be counted [31].

The next section provides an overview of two price control mechanisms employed in the pharmaceutical sector.

### Drug price regulation

#### Price regulation

Drug price regulation is most often exercised through insurers’ limiting the amount they will pay for a given drug. Cost-effectiveness analysis is essentially a form of price regulation; however in many countries there are supplementary rules about pricing. Such price regulation frequently uses some comparative, or yardstick, mechanisms. Belgium, France, Italy and the Netherlands use a direct yardstick method to evaluate orphan drug prices, directly comparing domestic prices to those issued in other countries [26]. In Canada, the Patented Medicine Prices Review Board (PMPRB) uses two tools to set a ceiling price. For new drugs offering a substantial improvement over existing therapies, the Canadian price is not allowed to exceed the median price for the same drug in seven other countries. This is only useful if the other countries don’t use the same comparative mechanism, and if the prices measured in those countries are real prices. Comparative price setting is leading many companies to set high nominal prices with hidden rebates so that comparisons won’t limit pricing elsewhere. The PMPRB uses a different tool for new drugs that offer little or no therapeutic advantage: their price is limited to be no higher than the price of comparable drugs [32].

While the price cap set under the PMPRB is compulsory, this regulatory oversight is separate from the decisions of drug plans (generally administered by the provincial governments in Canada). As such, the PMPRB price cap provides only limited price protection, with the majority of price/coverage decisions dictated by the cost effectiveness and *ad hoc* orphan drugs exceptions (discussed above) made by provincial regulators/drug plans.

### Rate of return and cost-yardstick regulation: U.K. PPRS

The use of ROR is uncommon in drug price regulation; however the United Kingdom’s Pharmaceutical Price Regulation Scheme (PPRS) has included a profit control scheme that bears some resemblance to a ROR model. The PPRS profit control scheme is rarely binding due to the existence of a separate value-based price cap (similar to that discussed above). Yet, despite its redundancy, the PPRS profit control mechanism includes some important features that distinguish it from the conventional application of ROR to utilities.

The PPRS agreement for 2005 to 2009 outlined an upper limit on pharmaceutical firm’s profits. This profit constraint can be represented by equation (2):

(2) $$\mathrm{Profits}\leq \max\left\{\left(29.4\%•\mathrm{Total}\;\mathrm{Capital}\right),\left(8.4\%•\mathrm{Total}\;\mathrm{Sales}\right)\right\}$$

Note that the constraint implies a firm can earn up to a 29.4% return on capital. In a meta-analysis of cost estimates Morgan et. al. [23] indicate that recent cost of capital estimates for pharmaceutical firms are around 11%, in the same range as the regulated rates of return for National Energy Board regulated pipelines in Canada. This suggests that the allowed rate of return under the PPRS is much higher than would be necessary to fairly compensate firms.

Unlike the standard utilities application of the ROR model described above, the PPRS does not include provisions for periodic hearings to gather information on the cost elements of regulated firms. Instead the PPRS imposes pre-determined values for cost elements in its profit calculations. Fixed allocations are set for research and development costs, marketing costs and information costs.

These cost allocations act as a cap on the costs that a pharmaceutical firm can claim against its revenues when calculating the revenue cap. Marketing and information costs can only be claimed if incurred domestically (i.e. - in the U.K.) while Research and Development allocations are made for costs incurred domestically or abroad. Allocations for marketing and information costs have a pre-determined cap while research and development costs are capped at a specific portion of the firm’s total sales.

The OFT report on the 2005–2009 PPRS acknowledges that, by its very nature, cost-based regulation is set up to reward inputs rather than outputs [33]. That is, a low-value product produced at high cost will fetch a higher regulated price than a high-value product produced at a low cost, distorting firms’ investment choices [34]. In addition to reducing the costs of regulation and the information required by the regulator, cost allocations limit the regulated firm’s ability to over-report or inflate costs, largely mitigating this issue.

The price cap and ROR models discussed focus on protecting consumers/payers by limiting the prices for drugs. While a suitably chosen regulated fair rate of return should be effective in maintaining an incentive for orphan drug development, the implementation of the U.S. Orphan Drug Act has demonstrated that regulated market exclusivity plays a substantial role and may be essential in incentivising development. Even though the U.S. case does not represent a price control scheme we cannot ignore it in discussing effective policies for orphan drugs pricing.

### No price controls: U.S. Orphan Drug Act (ODA)

The U.S. ODA focuses primarily on creating incentives for pharmaceutical firms to invest in orphan drugs development. Prior to its introduction, development of drugs for rare diseases was seen as unprofitable due to the large fixed cost of development and small patient numbers as discussed above. The ODA is focused on reducing or offsetting R&D costs and increasing the expected revenues associated with marketing an orphan drug [35].

To achieve the latter goal, the ODA stipulates a seven year market exclusivity period for any orphan drug approved by the U.S. Food and Drug Administration (FDA). During the period of exclusivity the FDA will not approve another drug for the same indication without consent of the “exclusive” drug’s manufacturer. A single manufacturer may have one or more “exclusive” drugs approved for a single indication, and any single drug can have exclusivity for a number of indications, but no two manufacturers can have exclusivity for a single indication without consent of the originally approved orphan drug’s manufacturer [35].

The EU enacted a set of similar policies in 2000 including development subsidies, other financial and bureaucratic benefits and a ten-year period of exclusivity for Orphan Drugs [26]. While subsidies and other direct financial benefits no doubt provide increased development incentives this exclusivity period (7 year U.S. and 10 year E.U.) is widely regarded as the most sought after incentive introduced by the ODA and EU policies, consistent with empirical evidence showing that potential competition (prior to patent expiration) is the largest deterrent to investment in a new drug [36].

The FDA approved only ten orphan disease treatments prior to the establishment of the ODA and has approved hundreds since [37] and the orphan drug market is now growing faster than the conventional drug market. However, the imposition of a seven-year exclusivity period (while an integral part of the ODA’s incentive system) without associated price controls creates an opportunity to exercise market power that is challenging to all insurers, state or private. The next section further details how the ROR model as applied to utilities can be adapted for orphan drug price control.

### A rate of return model for orphan drug price regulation

Due to the defined small market for orphan drugs and the related lack of suitable markets against which to yardstick, direct yardsticking of orphan drugs prices is likely infeasible. Applying a cost-effectiveness measure to orphan drugs has also all but been ruled out as a sufficient approach since orphan drugs are typically too costly. The similarities in market and cost structures between orphan drugs and utilities illustrated in section 2 indicate that regulatory methods which have proven effective for utilities have merit in application to orphan drugs. The existing precedent under the U.K. PPRS of ROR style profit controls is also encouraging despite the fact that these controls have proven largely redundant.

As discussed, the risk structure of orphan drugs development is more complex than a comparable undertaking in a utility. As such, care must be taken in identifying an appropriate regulated rate of return for orphan drugs especially since the cost of capital associated with orphan drug development likely varies depending on the stage of production.

Since risk is a significant portion of the cost of capital we would suggest that multiple rates of return be applied to the firm’s sunk capital investment at different stages of development. A high risk, high rate of return should be applied to the sunk costs allocated for years prior to initial regulatory approval. Following regulatory approval the rate of return would then transition to lower level representing the reduced ex-post risk following approval.

In effect, the initial high rate of return would be applied retroactively to the pre-approval costs associated with orphan drug R&D, thus essentially setting or modifying the initial rate base for the application of rate of return. As a basic example, assume that the firm is allocated a pre-clinical cost C_1_ at time T_1_ and that the cost allocated to conduct clinical trials C_2_ is incurred at time T_2_. Regulatory approval occurs at time *V* such that V > T_1_ > T_2_. The initial rate base would then be given by equation (3):

(3) $${\left(\mathrm{Rate}\;\mathrm{Base}\right)}_{t=V}=C_{1}•{\left(1+r_{H}\right)}^{V-T_{1}}+C_{2}•{\left(1+r_{H}\right)}^{V-T_{2}}$$

where r_H_ is the high risk first stage rate of return. Following regulatory approval, the effective revenue cap for an orphan drug would then be calculated as in equation (4):

(4) $$\begin{array}{l} \mathrm{Revenue}\leq \left[\left(\mathrm{Rate}\;\mathrm{Base}‒\mathrm{Accumulated}\;\mathrm{Depreciation}\right)•r_{L}\right]\\ \;+\left[\mathrm{Other}\;\mathrm{Costs}\right]+\left[\mathrm{Depreciation}\;\mathrm{Expense}\right] \end{array}$$

where *r*_*L*_ is the low risk second stage rate of return. In this formulation, the “Rate Base” would be a measure of sunk costs (R&D). “Other Costs” refers to the continuing variable costs incurred by the firm (i.e. - those costs associated with marketing and actual production of the drug etc.). The last term “Depreciation Expense” refers to the contribution towards paying down the principle liability associated with the allocated costs in the rate base. The depreciation expense is discussed in more detail below.

The complex risk structure for orphan drugs implies that cost allocations (like those employed under the PPRS profit control scheme) are more appropriate than the “used and useful criterion” (applied in utilities jurisdictions) when determining costs. By their nature, R&D expenditures in pharmaceutical jurisdictions are stochastic in productivity, in contrast to sunk capital investments in utilities jurisdictions. The use of fixed cost allocations rather than a formal assessment of costs will also avoid the cost-padding issue inherent in cost-based pricing mechanisms.

To appropriately set these cost allocations regulators/insurers should commit to a periodic review of industry-wide costs potentially punctuated by regular multi-party cost hearings used to determine appropriate deemed costs or cost caps. Deemed costs produced by these hearings would be based on industry costs, including both big pharma and biotechs, and would therefore maintain much if not all of a firm’s incentives to minimize costs under rate of return style regulation. Since the regulators and insurers are not privy to detailed information on the cost structure of orphan drug firms, the use of cost allocations based on a broad industry overview represents both a desirable, and potentially the most accurate feasible, mechanism to set cost inclusions in the rate base. The cost allocations should reflect the average cost to develop and bring to market a new orphan drug, accounting comprehensively for the risk of failure and the cost of capital. There is a lively debate about the true costs of R&D [23,25]. We do not attempt in this paper to describe exactly how to measure the average cost of drug development; this would be the task of the cost hearings.

The depreciation expense would largely be dictated by the size of the rate base at the time of approval and the duration of exclusivity granted. The depreciation expense needs to be set sufficiently high to ensure that the firm is able to recoup the total value of its deemed sunk costs (the total value of the rate base) prior to expected generic entry [38]. There is a trade-off in setting the depreciation expense and the period of exclusivity. A short exclusivity period implies a high depreciation expense and high prices with the benefit being an accelerated move to potential generic competition. Conversely, a long exclusivity period implies a low depreciation expense and low regulated prices with the cost being a delayed move to potential generic competition.

An insurer could calculate the amount it needed to contribute to the revenue requirement on the basis of its income share among OECD countries. Consider a simple example of how this might work for the United Kingdom. Suppose that it is found that the average new orphan drug has capital costs of $1bn. The United Kingdom’s share of this is approximately 5% (calculated as its share of OECD GDP). So the average drug development cost attributable to the UK is in the range of $50 m. Given 10 years of exclusivity enabled by patent protection, this implies that a firm would have to earn roughly $8 m per year above its operating costs to recoup the UK share of the average costs of development. If there were 200 patients using the product in the UK, that would suggest that the price per patient should allow for the assessed production and distribution costs, plus an additional $40,000 to pay for the pro rata R&D costs.

Formal regulatory hearings (in which firms, and interested intervener groups file evidence and formal arguments) would likely be effective in determining the appropriate exclusivity period (and corresponding depreciation rate) and cost allocations. In our estimation, such hearings should also prove to be an efficient forum for the discussion and determination of reasonable regulated rates of return. Since the regulated rate of return should allow some cross-subsidization towards the costs of failed attempts, it is necessary that firms and all other interested parties be consulted to provide evidence supporting a formal regulated rate of return. We would foresee this evidence taking the form of statistical analysis on failed development attempts and costs presented on behalf of the pharmaceutical firms and subject to substantial review by other interested parties including patient groups and insurers. Determining an effective risk premium in this manner is a contentious subject in its own right, and the regulated rate of return should represent a balance between the interests of firms and their shareholders and those of insurers and their patients. In any case, compared to an approach with confidential pricing in the context of a product listing agreement, this approach is more transparent and therefore less likely to lead to free-riding by countries.

An important question is whether the model we propose here would sustain the existing level of R&D in orphan drugs. If the only effect of using a rate of return calculation is to reduce expenditures, it is evident that it would discourage R&D. However, most governments deal with binding constraints in drug expenditures, and increased spending on one product is likely to reduce spending on another. A rate of return model could help insurers to achieve a better, more predictable spread of expenditures across products. That is, by reducing expenditures on orphan drugs that are earning very high rates of return, there would be more budget space for other orphan drugs. In this case, there is no reduction in expected revenues to support R&D, but a more even allocation of revenues across products, reducing risks for companies.

## Conclusion

Current price comparisons and cost-effectiveness measures, while adequate for pricing drugs for common diseases, have proven problematic when applied to orphan drugs. The attempts by firms to argue for high prices based on high average costs, combined with the apparent similarities between cost structures in the utilities sectors and pharmaceutical production shows that a form of cost based regulation should be effective in limiting the prices paid for orphan drugs while still fairly compensating firms.

As such, in cases where the insurer wishes to pay for a drug that does not meet the cost-effectiveness standard, we believe it is worth exploring the use of a rate of return model of regulation with the following modifications,:

1. The use of deemed rather than directly observed costs in calculating the rate base used under rate of return regulation. Periodic industry-wide hearings would be conducted at fixed intervals to review these deemed costs.

2. A fixed period of exclusivity for a drug with one or more indications following the precedent set under the U.S. Orphan Drug Act. This exclusivity period would be accompanied by an appropriate depreciation expense in the cost calculations such that the firm recoups its initial investment prior to potential generic competition.

3. A multi-stage approach for the regulated rate of return, with higher regulated rates of return for costs incurred prior to regulatory approval (reflecting the high risk of pre-approval investments) and a lower rate of return in the period after regulatory approval (reflecting the significantly reduced risk of investment following initial approval).

While the implementation of any such system requires more investigation to resolve specific practical issues, such work is warranted given the success of the rate of return model in application to utilities jurisdictions and the identified issues with extending common pharmaceutical price cap and yardstick methodologies to the case of orphan drugs. It has been suggested that orphan drugs markets could greatly benefit from additional transparency in pricing mechanisms [8]. At the very least, even a loose cost-based regulatory framework resembling the one we outline above should greatly improve transparency and can potentially benefit orphan drugs developers/manufacturers as well as insurers and their patients.

This proposal does not link the “value” of a new drug to its price; instead, price would be, in effect, a function of the rarity of the disease or condition treated. The reason for excluding value is that we are considering drugs with unacceptable cost-effectiveness ratios, and which would be more or less automatically excluded from insurance if the only rule used were cost-effectiveness. Despite this, these drugs are sometimes included in insurance formularies. But if the insurer throws out cost-effectiveness as a tool, what rule is left? Our proposal provides a principled way to determine a reasonable price for orphan drugs that fail cost-effectiveness tests, but offer compelling medical value.

## Competing interests

Hollis: Vice-President (unpaid) of Incentives for Global Health.

## Authors’ contributions

GKF and AH conceived and wrote the article together. Both authors read and approved the final manuscript.
